# A method based on light scattering to estimate the concentration of virus particles without the need for virus particle standards^☆^

**DOI:** 10.1016/j.mex.2015.02.003

**Published:** 2015-02-16

**Authors:** István Makra, Péter Terejánszky, Róbert E. Gyurcsányi

**Affiliations:** MTA-BME “Lendület” Chemical Nanosensors Research Group, Department of Inorganic and Analytical Chemistry, Budapest University of Technology and Economics, Szt. Gellért tér 4, Budapest 1111, Hungary

## Abstract

Most often the determination of the concentration of virus particles is rendered difficult by the availability of proper standards. We have adapted a static light scattering based method for the quantification of virus particles (shown for poliovirus) without the need of virus particle standards. Instead, as standards, well-characterized polymeric nanoparticle solutions are used. The method is applicable for virus particles acting as Rayleigh scatterers, i.e., virus particles with equivalent diameters up to ca. 1/10th of the wavelength of the scattered monochromatic light (∼70 nm diameter). Further limitations may arise if the refractive index of the virus is unavailable or cannot be calculated based on its composition, such as in case of enveloped viruses. The method is especially relevant for preparation of virus particle concentration standards and to vaccine formulations based on attenuated or inactivated virus particles where the classical plaque forming assays cannot be applied. The method consists of:

• Measuring the intensity of the light scattered by viruses suspended in an aqueous solution.
• Measuring the intensity of the light scattered by polymeric nanoparticles of known concentration and comparable size with the investigated virus particle.
• The concentration of virus nanoparticles can be calculated based on the two measured scattered light intensities by knowing the refractive index of the dispersing solution, of the polymer and virus nanoparticles as well as their relative sphere equivalent diameters.

## Graphical abstract

**Figure:**
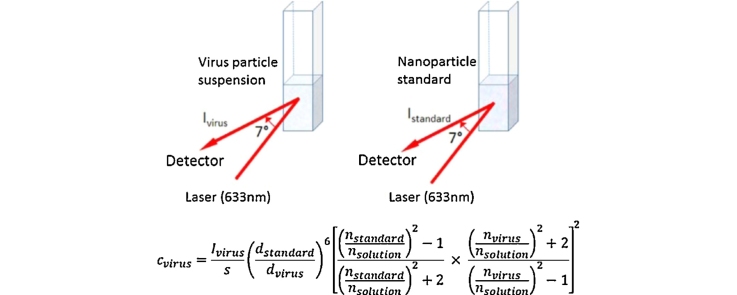

## Method details

The method is based on the Rayleigh approximation which states that if the diameter of the light scatterer is smaller than ca. 1/10th of the wavelength of the light source then the scattered light intensity depends on the scatterer concentration, diameter, and its refractive index relative to the solution refractive index. Thus by having two types of nanoparticles (e.g., latex and virus particles) which have different, but known diameters and refractive indices, one can determine the concentration of one of them (e.g., virus) if the concentration of the other (e.g., latex) is known by measuring the respective scattered light intensities. By this the concentration of the virus is referenced against the concentration of a conveniently chosen nanoparticle standard without the need for virus particle concentration standards that are very difficult to obtain. The expression for the calculation of virus particle concentration was adapted from Vysotskii et al., [1]:

(1) $$c_{\text{virus}}=c_{\text{standard}}\frac{I_{\text{virus}}}{I_{\text{standard}}}{\left(\frac{d_{\text{standard}}}{d_{\text{virus}}}\right)}^{6}{\left[\frac{{\left(n_{\text{standard}}/n_{\text{solution}}\right)}^{2}-1}{{\left(n_{\text{standard}}/n_{\text{solution}}\right)}^{2}+2}\times \frac{{\left(n_{\text{virus}}/n_{\text{solution}}\right)}^{2}+2}{{\left(n_{\text{virus}}/n_{\text{solution}}\right)}^{2}-1}\right]}^{2}$$

where *c*_virus_ is the virus concentration, *d*_virus_ and *d*_standard_ are the virus and the standard nanoparticle diameter, *I*_virus_ and *I*_standard_ are the scattered light intensities stemming from the virus and the nanoparticle standards, *n*_solution,_ *n*_standard_ and *n*_virus_ are the refractive indices of the solution, the standard particles and the virus, respectively. The expression is valid for virus and standard nanoparticles that are Rayleigh scatterers.

We have brought Eq. (1) to a more accurate form by replacing the ratio of *I*_standard_/*c*_standard_ with the slope of the line fitted to the linear range of the standard particle intensity–concentration plot, *S* (*S* = Δ*I*_standard_/Δ*c*_standard_):

(2) $$c_{\text{virus}}=\frac{I_{\text{virus}}}{s}{\left(\frac{d_{\text{standard}}}{d_{\text{virus}}}\right)}^{6}{\left[\frac{{\left(n_{\text{standard}}/n_{\text{solution}}\right)}^{2}-1}{{\left(n_{\text{standard}}/n_{\text{solution}}\right)}^{2}+2}\times \frac{{\left(n_{\text{virus}}/n_{\text{solution}}\right)}^{2}+2}{({n_{\text{virus}}/n_{\text{solution)}}}^{2}-1}\right]}^{2}$$

The method proceeds with determining the parameters involved in Eq. (2).

1. Scattered light intensities: the intensities of the light scattered by the virus and latex nanoparticle solutions (the latter is used as standard) can be measured using a dynamic light scattering (DLS) instrument, which has the option to determine the static light scattering. In both cases, it is recommended to check the linearity of the scattered light intensity as a function of the respective particle concentrations and proceed with concentrations that are within the linear range.
2. Virus related parameters: the virus diameter is generally well known from literature or can be determined by electron microscopy (EM) [2], nanoparticle tracking analysis (NTA) [3], or resistive pulse sensing (RPS) [4]. The refractive index of viruses are however more difficult to be retrieved from the literature, but can be estimated by calculation as it will be shown later in the detailed methodology.
3. Nanoparticle standard related parameters: as standard we propose well-characterized commercially available spherical latex nanoparticles with diameters close to that of the virus particles. The choice of latex nanoparticles as standard is motivated by the availability of all their relevant characteristics: concentration, refractive index and diameter.
4. Solvent/dispersant related parameters: the refractive index of the dispersant can be found tabulated in databases (e.g., Handbook of Chemistry and Physics) or can be readily measured using a refractometer.

## Determination of the scattered light intensity by the virus and nanoparticle solutions

### Determination of the slope of the scattered light intensity vs. latex nanoparticle concentration curve

**Fig. 1:**
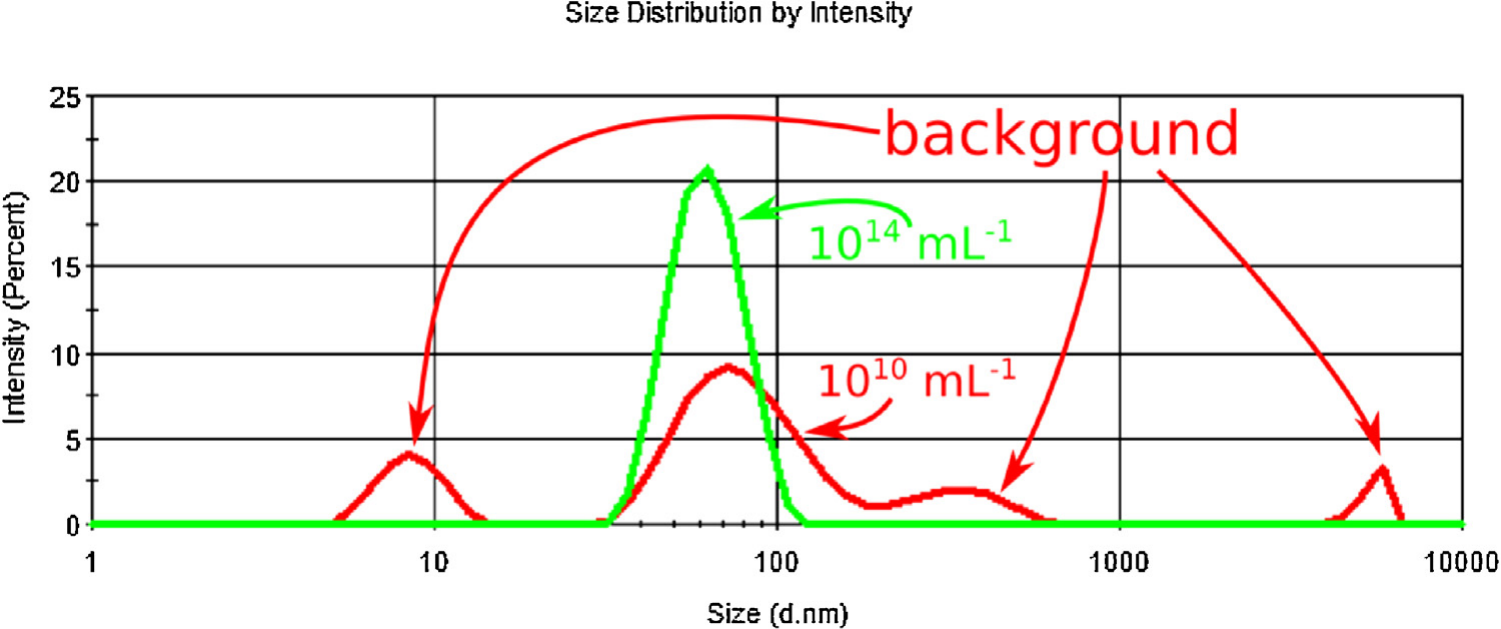
Intensity distribution plot of the 45 nm diameter nanoparticle standard at 10^14^ (green) and 10^10^ (red) mL^−1^ concentrations. It is apparent that at the lower concentration the scattered light intensity from other particles (e.g., impurities) becomes significant and the total light intensity needs to be corrected with the percentage stemming from the standards. At higher concentration however the peak of the standard particles is the dominant one and the total intensity of the scattered light can be used for calculation.

1. Measure the scattered light intensity for different concentration latex nanoparticles. It is advantageous to measure the backscattered light intensity instead of the forward scattering. Large (typically micron sized) debris particles scatter less light in the backward direction. Therefore, measuring the forward backscattered light the contribution of the background debris to the scattered light intensity is minimized.
2. As the solution may contain particles that differ from the standards but also scatter light, one needs to calculate the scattered light intensity stemming only from the standard nanoparticles. For this first the size distribution of the particles in the solution should be measured by DLS. The results should be plotted as intensity percent (intensity percent vs. size) and the peak corresponding to the standard particles should be identified (Fig. 1). The area of the peak corresponds to the percent of the intensity of the scattered light stemming from the nanoparticle standard. Multiplying with this percentage value the total intensity of the scattered light (stemming from all particles in the solution) results in the light intensity scattered by the standards. For the specific case of using the Malvern Zetasizer Nano ZS see the procedure detailed in the Supplementary information.
3. Plot the scattered light intensity as a function of particle concentration on a log–log scale and determine its linear range. Fit a line in *I*_standard_ = *s* × *c*_standard_ + *b* shape to the linear range and determine its slope (*s*). Note that while a line fitted in linear coordinates will be straight in case of a log–log scale this is not necessarily the case, i.e., will be a line only if *b* = 0.

### Determination of the scattered light intensity of the virus sample

**Fig. 2:**
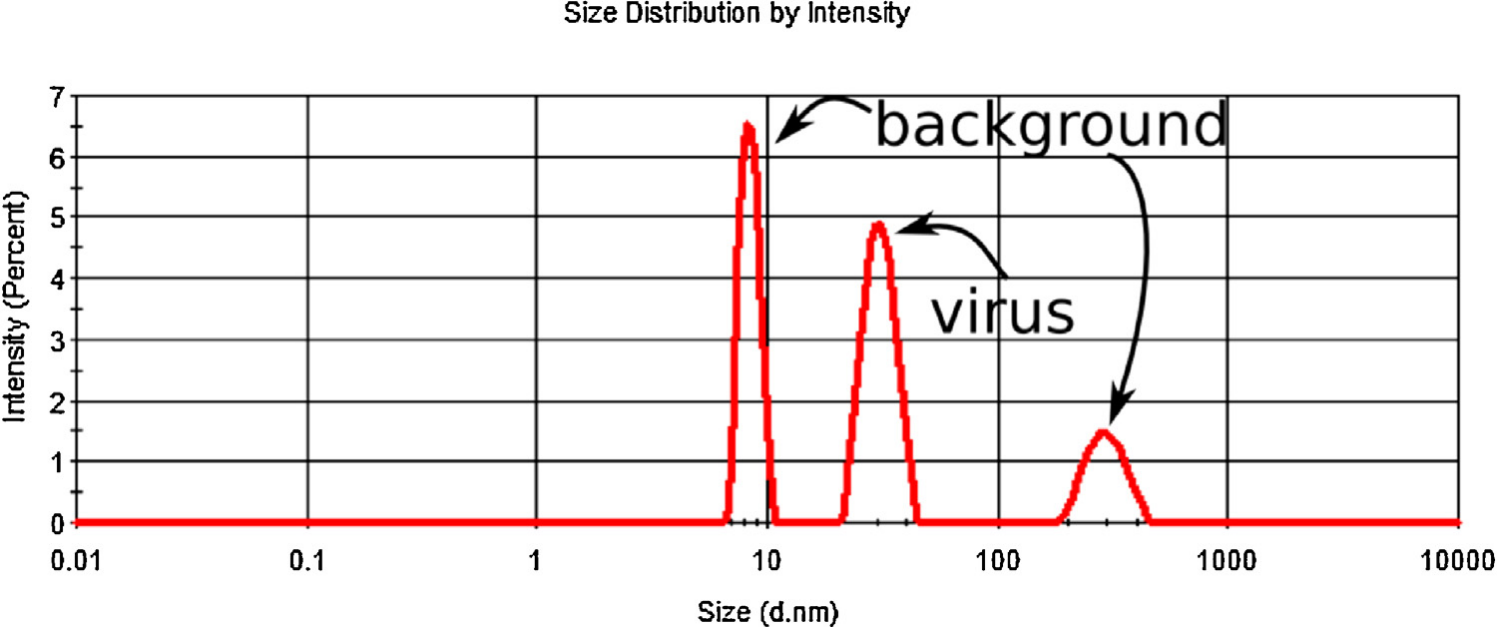
A typical intensity distribution plot of the poliovirus sample showing peaks at ca. 31, 8 and 297 nm diameter. The peak corresponding to the virions is the one centered at ca. 31 nm (the second peak).

The procedure is the same for steps 1–3 as for the standard nanoparticles but determining the light scattered at a single concentration level is sufficient. Still a simple dilution experiment is recommended to check whether the sample concentration is in the linear range of the method, i.e., the intensity of scattered light scales linearly with the dilution. Note that step #3, to determine only the fraction of the total scattered light intensity stemming from virus particles is especially important as virus particle solutions unlike polymeric nanoparticle standards may contain other particles that scatter the light (if insufficiently purified) (Fig. 2).

## Determination of the diameter representative of the whole particle distribution

Ideally one can determine a diameter that represents the whole standard or virus particle distribution in terms of light scattering. As the scattered light intensity scales with $d_{\text{part}}^{6}$, larger particles will have more statistical weight so that a simple arithmetic mean cannot be used. Having a particle size distribution stemming from EM or RPS which have minimal artificial distribution broadening, the so called scattering mean diameter can be calculated as

(3) $$d_{\text{mean}}^{\text{scatter}}=\sqrt[{6}]{\frac{1}{n}\sum_{\text{i}=1}^{n}d_{\text{i}}^{6}}$$

where *d*_i_ are the particle diameters, and *n* is the number of particles in the distribution. If this information is not available one can approximate the scattering mean diameter as the mean diameter of the size distribution stemming from another nanoparticle sizing method or as the mean diameter provided by the manufacturer.

## Determination of the refractive index of the virus

The preferred method is to use values previously reported in literature, but because of their limited availability they can be estimated by one of the following methods.

a) If the specific volume (or density) and amino acid composition of the virus is known then the refractive index of the virus should be calculated as described by McMeekin et al. [5], [6], [7] Briefly, by knowing the molar refractivity of the amino acids (*R*_a_, [mL/mol]) and knowing their mole or mass fraction in the virus, a weighted molar refractivity (*R_v_*, [mL/mol]) can be calculated for the virion. To do this, first the molar refractivity of each amino acid needs to be converted to molar refraction per gram by using:

(4) $$R_{\text{ag},\text{i}}=\frac{R_{\text{a},\text{i}}}{M_{\text{a},\text{i}}}[\text{ml}/\text{g}]$$

where *M*_a,i_ is the molecular weight of amino acid “i”. Then the molar refractivity per gram for the virus is calculated using:

(5) $$R_{v}=\sum_{\text{i}}R_{\text{ag},\text{i}}w_{\text{i}}$$

where *w*_i_ is the mass fraction of amino acid “i”.

Finally the refractive index of the virus is calculated from Eq. (5) by using the Lorentz–Lorenz formula:

(6) $$n_{v}=\sqrt{\frac{2R_{v}+{\bar{v}}_{v}}{{\bar{v}}_{v}-R_{v}}}$$

where ${\bar{v}}_{v}$ [ml/g] is the specific volume of the virus as obtained from the literature.

b) If the only parameter that is known is the specific volume (or density) of the virus, the refractive index may be calculated as the refractive index of a protein layer with a specific volume equal to that of the virus:

(7) $$n_{v}=n_{s}+\frac{1}{{\bar{v}}_{v}}\frac{\text{d}n}{\text{d}c}$$

where *n*_s_ is the refractive index of the solvent (1.33 for water, 1.332 for PBS as predetermined in the Zetasizer software) and $\frac{\text{d}n}{\text{d}c}$ is the refractive index increment of the virus (approximated as 0.1888 ± 0.0025 ml/g which is valid for human proteins above 100 kDa molecular weight [7]).

c) The third approach is to approximate the refractive index of the virus with the refractive indices of similarly sized viruses found in the literature.

## Calculation of virus concentration

The virus concentration can be calculated using Eq. (2) after implementing all previously determined parameters.

## Validation of the method for poliovirus samples and latex nanoparticle standards

**Fig. 3:**
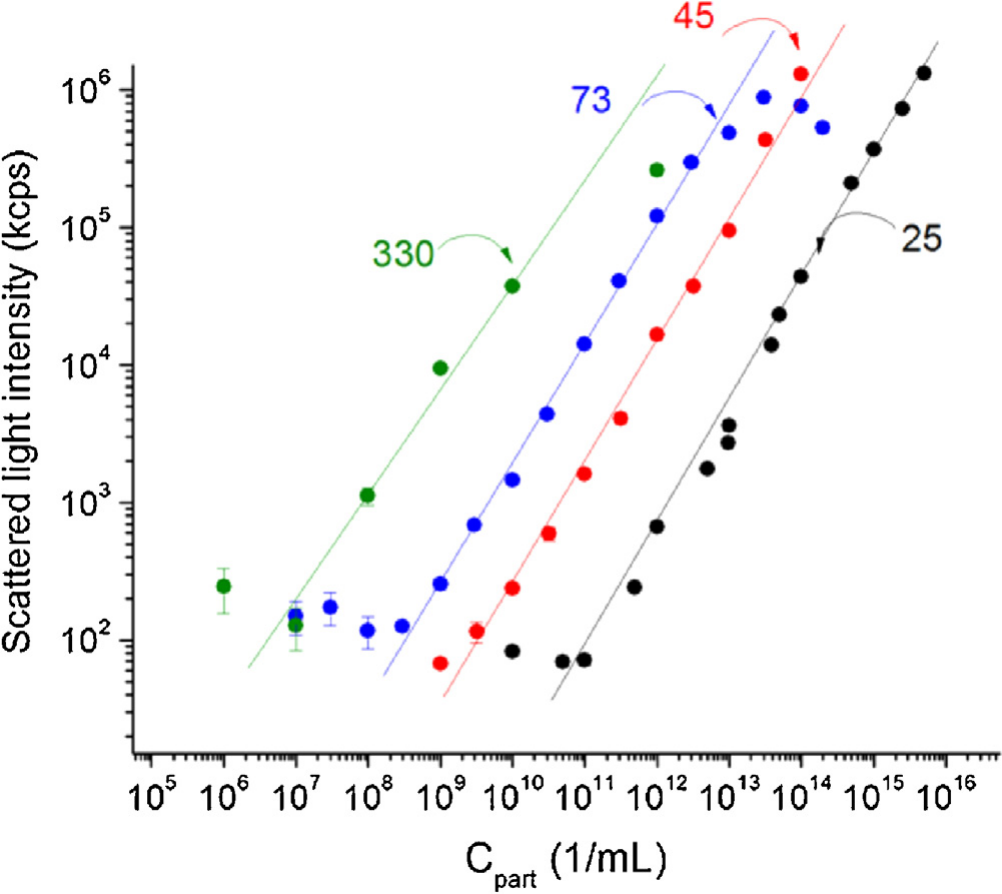
The scattered light intensity stemming from different sized standard nanoparticles as a function of their concentration (note the log–log scale). Lines are fitted to the linear range of the respective dependencies.

Fig. 3 shows the scattered light intensity (in kcps, kilo count-per-second) as a function of latex nanoparticle concentration in aqueous solution for various size nanoparticles. Given that most DLS instruments use red laser (633 nm in case of Zetasizer Nano ZS) the 25, 45 and 73 nm diameter nanoparticles can be considered as Rayleigh scatterers, while the 330 nm nanoparticle clearly exceeds the size limit. Owing to the strong ($d_{\text{part}}^{6}$) dependency of the scattered light intensity on the particle diameter the linear range of scattered light intensity vs. nanoparticle concentration curves is shifting towards lower concentration. The limits of the linear range at low concentrations where the scattered intensity from the particles falls below about 200 kcps is given by the detection limit of the instrument while at high concentrations multiple scattering events occur that will artificially lower the scattered light intensity reaching the detector. Fig. 3 shows clearly why it is recommended to check whether the concentration of the particle standard is in the linear range.

**Fig. 4:**
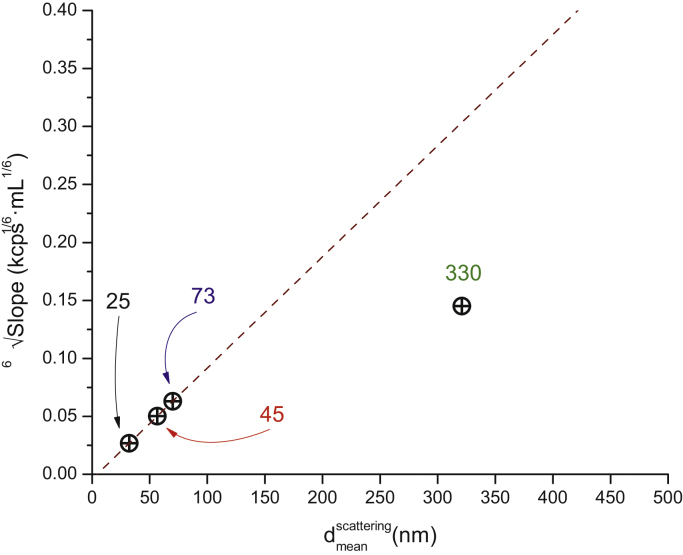
The 6th root of the slope of the lines fitted on Fig. 3 depend linearly on $d_{\text{mean}}^{\text{scatter}}$ as long as the particle diameter remains below of ca. 63 nm (1/10th of the illuminating laser wavelength). The fitted line is a guide for the eye.

The fact that the 330 nm diameter particle cannot be considered a Rayleigh scatterer becomes evident by plotting the 6th root of the slopes of the linear fits as a function of the particle diameter. Given that the nanoparticles are from the same material, i.e., they have the same refractive index, such a plot should be linear with a zero intercept. While the experimental data fall on a line for nanoparticles smaller or equal than 73 nm, this is clearly not true for the 330 nm diameter nanoparticle (Fig. 4).

**Fig. 5:**
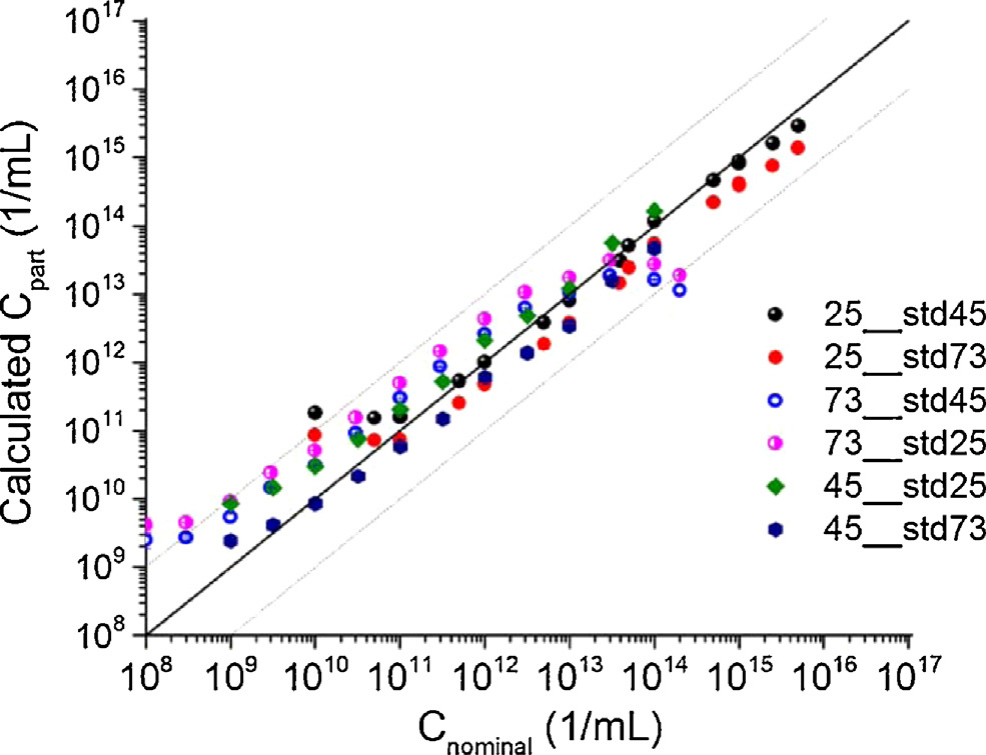
Calculated concentrations (Eq. (2)) of the latex nanoparticles with the use of other (different size) latex particle as standard (see legend). The solid line denotes the perfect correlation while the dashed lines are shifted by one order of magnitude up and down with respect to the perfect correlation.

The method was validated by comparing the nominal concentration values of the three different size nanoparticles with the values calculated with Eq. (2). For each size nanoparticles the concentrations diameters were calculated using the other two size nanoparticles as standards, e.g., the concentrations of different solutions of 25 nm diameter nanoparticles were calculated using 45 and 73 nm nanoparticles as standards. The results are shown in Fig. 5 and illustrates the accuracy of the method. Large discrepancies are apparent only where the linear dependence of the scattered light intensity vs. nanoparticle concentration is not valid.

In the following, we approximated the refractive index of poliovirus with the aforementioned methods. The value of *n*_v_ = 1.535 was found in the literature; however, this can be considered only as a lower approximation of the refractive index, because of the unknown water content of the virus crystal. [8]. The refractive index calculated based on the virus amino acid composition [9], [10] and specific volume (0.685 ml/g) [11] is *n* = 1.542 ± 0.005 while that based on specific volume and refractive index increment is *n* = 1.619 ± 0.004.

Because a significant part of the virus can stem from DNA or RNA (31.6 mass percent for poliovirus) [11] one must investigate if it is reasonable to treat the whole virus like a protein aggregate in terms of its light scattering properties. According to the literature, DNA films have a refraction index in the range 1.51–1.58, [12] which is close to the refractive indices determined by calculations assuming that the whole virus is made of proteins. So it is reasonable to approximate the whole virus as a protein regarding light scattering.

We approximated *n*_v_ = 1.55–1.67 for poliovirus using the refractive indices of bacteriophage MS2 which has a diameter of 25–30 nm and an icosahedral symmetry similar to the poliovirus [13].

Considering that protein refractive indices are in the range of 1.45–1.65 [5], [6], the values calculated by us seems valid and in the following we considered the *n*_v_ = 1.58 ± 0.04 range for the refractive index of poliovirus.

In case of enveloped viruses where the viral protein capsid is covered by a lipid envelope the methods based on calculations may become too complex and uncertain as the lipid bilayer has a refractive index of 1.48–1.49 [14]. In such a case relying on refractive index values of similar viruses or the target virus is the preferred method.

In the next step the concentration of the poliovirus samples was calculated using all three size latex nanoparticles as standard that where shown to be Rayleigh scatterers. The calculated virus concentrations are in rather good agreement with the previously measured (6.5 ± 1.8) × 10^12^ mL^−1^ by RPS measurements [4]. According to our calculations the virus refractive index should lie in the 1.68–1.80 range (depending on the diameter of standard particles) to obtain the same concentration as determined by RPS. The results along with the parameters used for the calculation are summarized in Table 1.

## Chemicals, materials and instruments

• Deionized water with 18.2 MΩ cm resistivity (Millipore).
• Phosphate buffered saline tablet (P4417, Sigma–Aldrich).
• Carboxylate-modified latex nanoparticles with nominal diameters (stock concentrations) of 330 nm (2 × 10^12^ mL^−1^), 73 nm (2 × 10^14^ mL^−1^), 45 nm (8 × 10^14^ mL^−1^), and sulfate-modified latex nanoparticles with nominal diameter 25 nm (10^16^ mL^−1^) purchased from Life Technologies Corporation (Carlsbad, CA).
• Inactivated poliovirus samples (Sabin-1 strain) provided by the National Center of Epidemiology (Hungary).
• BRAND^®^ UV cuvettes (center height 8.5 mm, chamber volume 70–850 μL, window width × height 2 mm × 3.5 mm) with 80–100 μl solution.
• 100 kDa Sartorius Vivaspin 500 centrifugal concentrator.
• Malvern Zetasizer Nano ZS particle analyzer with Zetasizer Software 7.01 (Malvern Instruments Ltd.) and a 4.0 mW He–Ne laser with a wavelength of 633 nm.
• Eppendorf 5430R centrifuge.

## Poliovirus sample preparation

The poliovirus stock solution was centrifuged through a 100 kDa Sartorius Vivaspin 500 centrifugal concentrator three times using an Eppendorf 5430R centrifuge at 13,000 × *g* and diluted with PBS +0.05% Tween-20 at pH 7.4 to 1/3 of the stock solution concentration.

## Measurement settings

The parameters set in the Malvern Zetasizer Nano ZS are listed but the measurements can be made using other dynamic light scattering (DLS) instruments that have the option to measure static light scattering.

• Measurement type: size.
• Material (latex nanoparticle standards): polystyrene latex, refractive index = 1.59, absorption = 0.01.
• Material (poliovirus): protein, refractive index = 1.45, absorption = 0.001.
• Dispersant (used for latex nanoparticle standards): water, temperature = 25 °C, viscosity = 0.8872 cP, refractive index = 1.33.
• Dispersant (used for poliovirus): phosphate buffered saline (constructed as “complex solvent” with the “dispersants manager” from 0.137 M NaCl, 0.0027 M KCl and 0.01 M Na_3_PO_4_), temperature = 25 °C, viscosity = 0.9103 cP, refractive index = 1.332.
• General options: use dispersant viscosity as sample viscosity.
• Temperature: temperature = 25 °C, equilibration time = 60 s.
• Cell: ZEN0040 disposable micro cuvette (40 μl).
• Measurement: measurement angle: 173° backscatter (NIBS default), measurement duration: automatic, number of measurements = 3, delay between measurements = 0 s.
• Advanced: positioning method: seek for optimum position, automatic attenuation selection: yes.
• Data processing (used with standard particles): general purpose (normal resolution).
• Data processing (used with poliovirus): protein analysis.

## Figures and Tables

**Table 1:** Calculated poliovirus concentration values along with the parameters used for their calculation, i.e., the experimentally determined scattered light intensity–concentration slopes (*s*), and the $d_{\text{mean}}^{\text{scatter}}$. Parameters with the same value for every calculation were $d_{\text{mean, polio}}^{\text{scatter}}=26.402\;\pm \;0.142\;nm$, *I*_virus_ = 2048 ± 56 kcps, *n*_standard_ = 1.59 ± 0.005, *n*_solution_ = 1.331 ± 0.001 and *n*_virus_ = 1.58 ± 0.04.

$d_{\text{part}}^{\text{nominal}}\left(\text{nm}\right)$	$d_{\text{mean}}^{\text{scatter}}\left(\text{nm}\right)$	*s* (kcps mL)	Poliovirus concentration (1/mL)
25	32.357 ± 0.225	(3.781 ± 0.175) × 10^10^	(2 ± 0.6) × 10^13^
45	56.597 ± 0.275	(1.604 ± 0.040) × 10^9^	(1.3 ± 0.4) × 10^13^
73	70.077 ± 0.273	(6.267 ± 0.961) × 10^8^	(1.2 ± 0.4) × 10^13^
