# Supplementary material for: A method based on light scattering to estimate the concentration of virus particles without the need for virus particle standards
Source: MethodsX. 2015 Feb 16;2:91–9. doi: 10.1016/j.mex.2015.02.003 (PMC4487338; doi:10.1016/j.mex.2015.02.003)
Supplement: Supplementary file 1 [file mmc1.docx]

## **Additional information**

**A method based on light scattering to estimate the concentration of virus particles without the need for virus particle standards**

**István Makra, Péter Terejánszky, Róbert E. Gyurcsányi^*^**

*MTA-BME “Lendület” Chemical Nanosensors Research Group, Department of Inorganic and Analytical Chemistry, Budapest University of Technology and Economics, Szt. Gellért tér 4, Budapest, 1111-Hungary*

**corresponding author:* [*robertgy@mail.bme.hu*](mailto:robertgy@mail.bme.hu)

## Calculation of the scattered light intensity stemming from the latex nanoparticles

As the solution may contain particles that differ from the standards/virions (Figure 1 and 2) but also scatter light, one needs to calculate the scattered light intensity stemming solely from the standards/viruses. To do this one can calculate the area of the peak representing the standard/virus particles which will give the percentage of the scattered intensity originating from the standards/viruses. Combining this percentage value with the total scattered intensity (that includes every particle in the solution) results in the scattered intensity value of the standards.

The operating software of the Zetasizer Nano ZS stores the total scattering intensity and the area percentage of the detected peaks on the intensity distribution plot for each measurement. For the total scattering intensity one should add the “Derived Count Rate” column to the record view by clicking on the following labels in the Zetasizer software:

Configure -> Record View Parameters -> Measurement category -> Size subcategory -> Derived count rate

For the area of an intensity peak (the percentage of the total scattered intensity) one should follow the sequence:

Configure -> Record View Parameters -> Measurement category -> Size subcategory -> Size peak

and choose:

- Transformation: Intensity
- Peak type: Area
- Peak: the index of the peak (peak indices are sorted by decreasing peak area and indexing starts at 1). This means that the peak that represents the largest part of the scattering intensity will have the index 1.


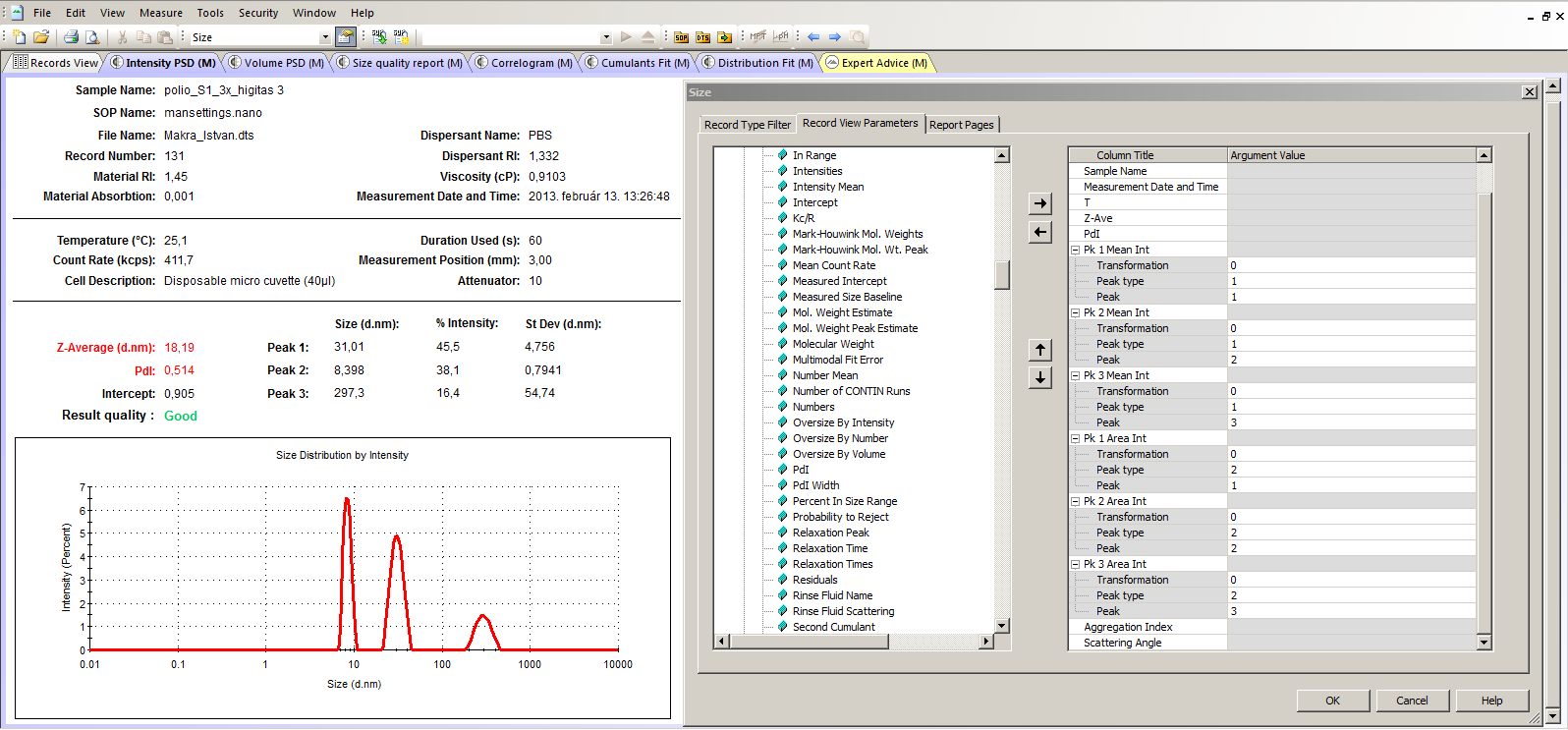


Figure S1: A snapshot of the Zetasizer software
